# Supplementary material for: Genetic determinants of daytime napping and effects on cardiometabolic health
Source: Nat Commun. 2021 Feb 10;12:900. doi: 10.1038/s41467-020-20585-3 (PMC7876146; doi:10.1038/s41467-020-20585-3)
Supplement: Supplementary file 1 — Supplementary Information [file 41467_2020_20585_MOESM1_ESM.pdf]

Genetic determinants of daytime napping and effects on cardiometabolic health

Dashti and Daghlas et al.

Supplementary Information

Supplementary Figure 1. (A) Q-Q plot for daytime napping genome-wide association study in the UK Biobank (*n* =452,633) using $-\log_{10}P$ values for all genotyped and imputed single-nucleotide polymorphisms (SNPs) passing quality control (BOLT-LMM mixed model association test *P* values) Q-Q plot shows the expected verses observed *P* values from association analysis. (B) Functional consequence of daytime napping variants on genes using FUMA. The histogram displays the proportion of SNPs which have corresponding annotation assigned by ANNOVAR. Bars are colored by $\log_2$enrichment relative to all SNPs in the reference panel. * *P* <0.05 (B).

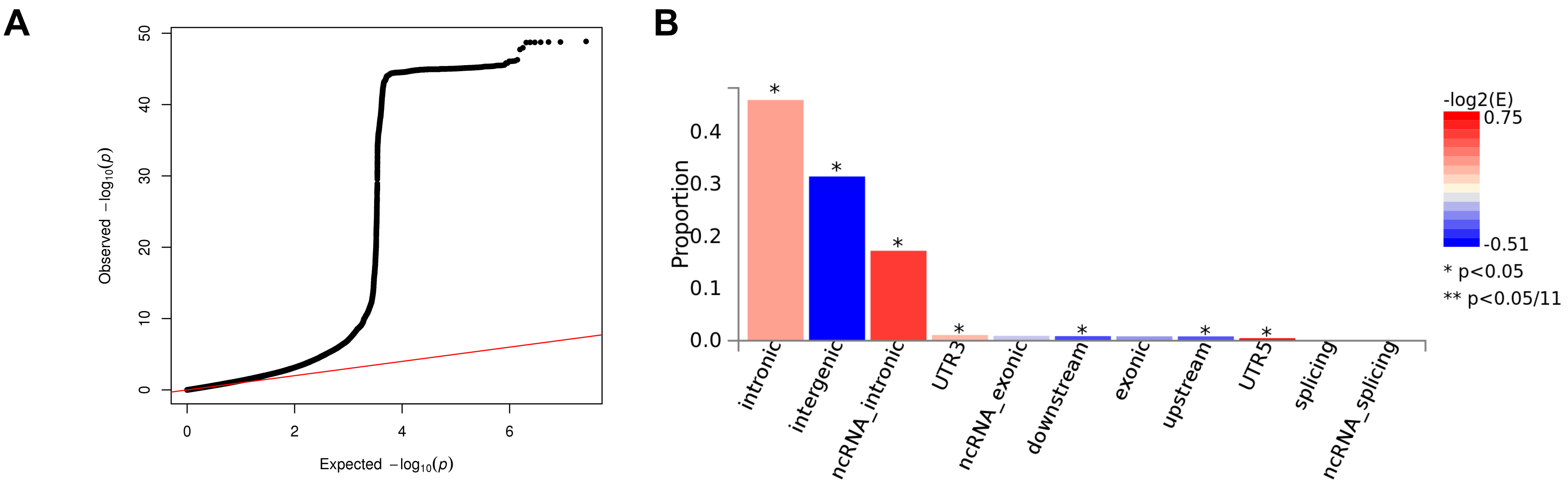

Supplementary Figure 2. Colocalization analysis reveals a shared causal variant associating with increased FNDC5 gene expression in skeletal muscle (*n* =706) and reduced napping liability (*n* =452,633). (A,B) Regional association plots for napping and FNDC5 gene expression at rs2786547 and variants within 400kb on chromosome 1. The y-axis shows the -$\log_{10}$ P value for each variant in the region, and the x-axis shows the genomic position. Each variant is represented by a filled circle, with the rs2786547 variant colored purple, and nearby variants colored according to degree of linkage disequilibrium (r^2) with rs2786547. The lower panel shows genes located in the displayed region and the blue line corresponds to the recombination rate. (C) Effect of T allele dosage on standardized FNDC5 gene expression in skeletal muscle (*n* =706). The white bar displays the point estimate for standardized gene expression, the boundaries of the dark bars display the interquartile range, the bounds of the green shading reflect the minima and maxima, and the width of the green shading reflects the probability density of the data at that expression value.

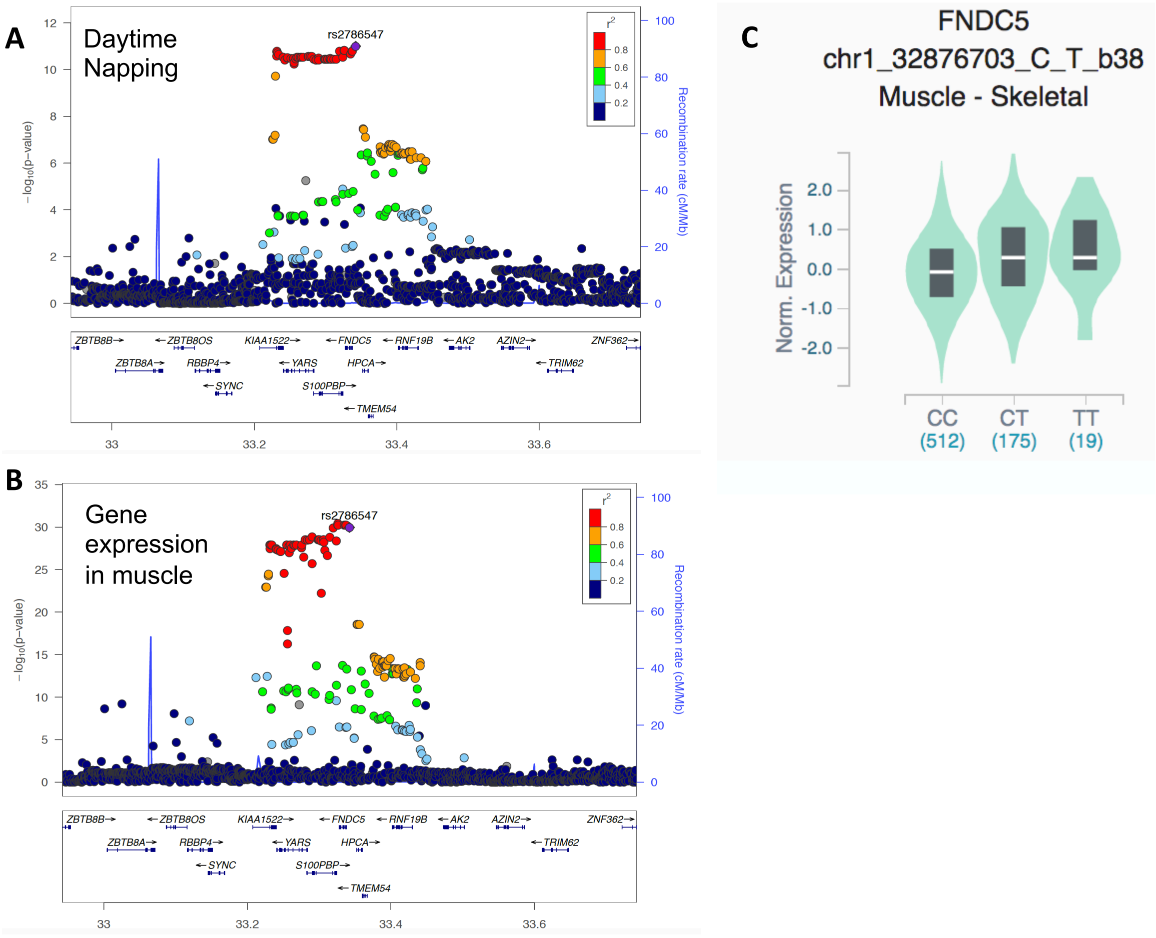

Supplementary Figure 3. Clustering of daytime napping variants using Bayesian nonnegative matrix factorization (bNMF) algorithm (A,B) and hierarchal clustering (C). * *P* <0.05

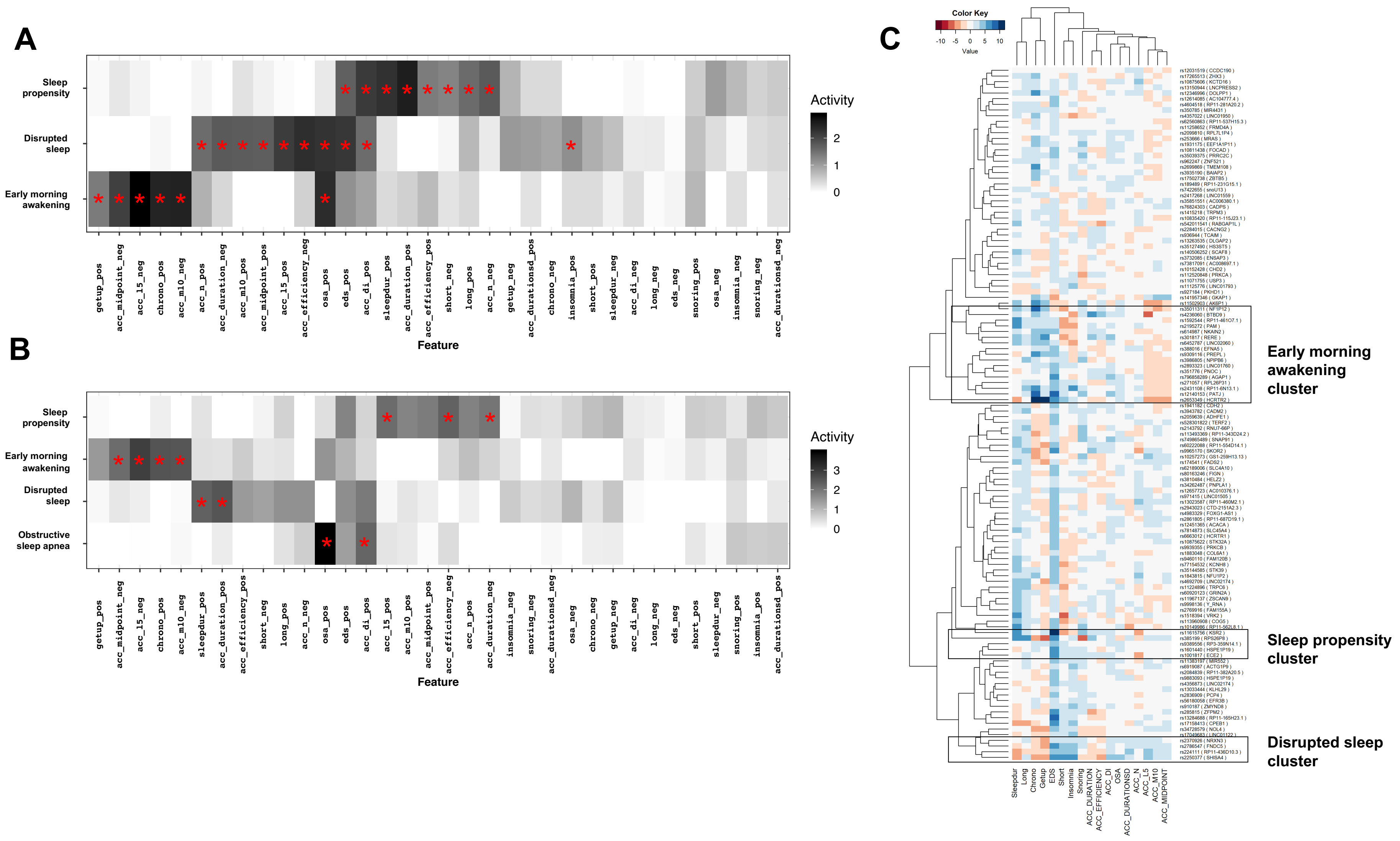

Supplementary Figure 4. Two-sample Mendelian randomization sensitivity analyses for the effect of daytime napping on systolic blood pressure, diastolic blood pressure, and waist circumference. Black boxes indicate the effect estimate and surrounding lines represent 95% confidence intervals. Abbreviations: DBP: diastolic blood pressure; IVW: inverse-variance weighted; SBP: systolic blood pressure.

Outcome	MR analysis	n SNPs	Beta [95% CI]	P value
SBP (sample 1)	IVW	106	0.34 [0.10, 0.58]	5.40E−03
	MR Egger	106	0.36 [−0.34, 1.07]	3.18E−01
	Simple median	106	0.36 [0.13, 0.60]	2.20E−03
	Weighted median	106	0.25 [0.01, 0.49]	4.07E−02
	MR−PRESSO	100	0.24 [0.04, 0.44]	2.30E−02
SBP (sample 2)	IVW	106	0.32 [0.11,0.53]	3.30E−03
	MR Egger	106	0.69 [0.04−1.34]	3.92E−02
	Simple median	106	0.19 [−0.04, 0.42]	1.11E−01
	Weighted median	106	0.41 [0.19, 0.64]	3.00E−04
	MR−PRESSO	103	0.29 [0.09, 0.48]	4.30E−03
DBP (sample 1)	IVW	106	0.14 [0.07, 0.22]	3.00E−04
	MR Egger	106	0.19 [−0.04, 0.42]	1.00E−01
	Simple median	106	0.15 [0.08, 0.23]	1.00E−04
	Weighted median	106	0.15 [0.08, 0.23]	1.00E−04
	MR−PRESSO	98	0.13 [0.07, 0.20]	1.00E−04
DBP (sample 2)	IVW	106	0.13 [0.06, 0.20]	2.00E−04
	MR Egger	106	0.23 [0.02, 0.44]	3.55E−02
	Simple median	106	0.08 [0.01, 0.14]	2.11E−02
	Weighted median	106	0.12 [0.06, 0.19]	4.00E−04
	MR−PRESSO	101	0.13 [0.07, 0.19]	5.01E−05
Waist circumference	IVW	73	0.28 [0.11, 0.45]	1.30E−03
	MR Egger	73	0.03 [−0.60, −0.66]	9.30E−01
	Simple median	73	0.23 [0.03, 0.42]	2.46E−02
	Weighted median	73	0.17 [−0.03, 0.36]	1.03E−01
	MR−PRESSO	73	0.28 [0.12−0.43]	8.30E−04

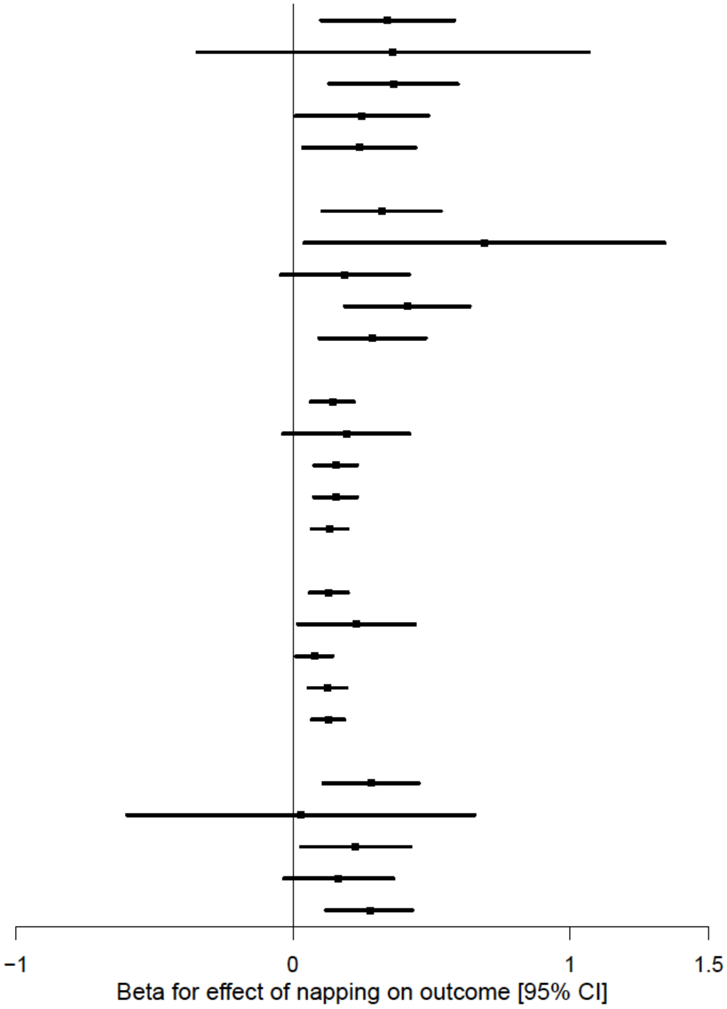

Supplementary Figure 5. Phenome-wide association study (PheWAS) of missense variants in *HCRTR1* and *HCRTR2* on 1,402 ICD-defined outcomes in UK Biobank reveals no phenome-wide significant disease associations. Each dot represents a phenotype, grouped into phenotype class by color. The y axis represents the $-\log_{10}$(p-value), with the dotted horizontal line representing the Bonferonni-corrected p-value threshold for phenome-wide significance.

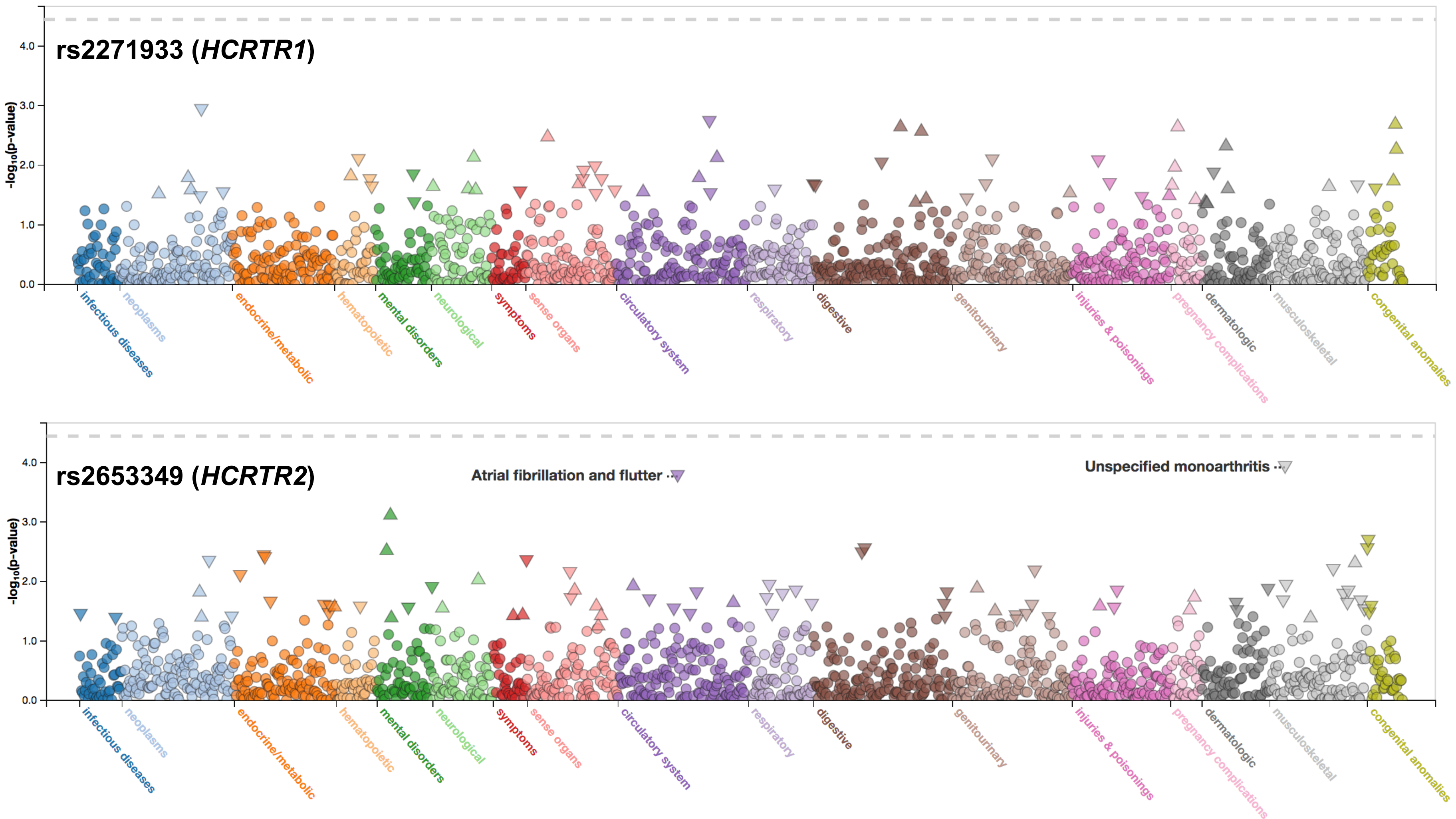

Supplementary Table 1. Descriptive table of UK Biobank participants of European ancestry included in daytime napping genome-wide association study.

	All participants (*n* =452,633)				Participants with *excellent* or *good* overall health (*n* =338,764) only			
	Self-reported daytime napping				Self-reported daytime napping			
**Characteristic**	Never/Rarely (*n* =255,746)	Sometimes (n =172,897)	Always (*n* =23,990)	*P* value	Never/Rarely (*n* =203,551)	Sometimes (n =121,411)	Always (*n* =13,802)	*P* value
**Sex (female), %**	59.0	50.0	33.9	< 0.001	60.4	51.3	33.0	< 0.001
**Age, y**	56.19 (8.06)	58.47 (7.77)	60.19 (7.38)	< 0.001	56.19 (8.07)	58.54 (7.78)	60.65 (7.23)	< 0.001
**24-hour sleep duration, hours**	7.08 (0.99)	7.24 (1.12)	7.69 (1.49)	< 0.001	7.12 (0.93)	7.28 (1.01)	7.60 (1.22)	< 0.001
**BMI, kg/m2**	26.90 (4.53)	27.97 (4.94)	28.68 (5.32)	< 0.001	26.38 (4.12)	27.20 (4.31)	27.52 (4.31)	< 0.001
**Waist Circumference, cm**	88.30 (12.96)	92.40 (13.63)	96.43 (14.22)	< 0.001	86.79 (12.17)	90.24 (12.54)	93.43 (12.51)	< 0.001
**Systolic blood pressure, mmHg**	137.03 (18.55)	139.19 (18.65)	140.57 (18.75)	< 0.001	136.66 (18.55)	139.07 (18.65)	141.22 (18.64)	< 0.001
**Diastolic blood pressure, mmHg**	81.91 (10.08)	82.55 (10.14)	82.88 (10.33)	< 0.001	81.57 (9.97)	82.35 (10.01)	82.93 (10.06)	< 0.001
**Townsend Deprivation Index**	-1.62 (2.90)	-1.32 (3.06)	-0.94 (3.24)	< 0.001	-1.81 (2.77)	-1.64 (2.87)	-1.48 (2.94)	< 0.001
**Sleep apnea diagnoses, %**	0.8	1.6	3.6	< 0.001	0.5	0.9	1.7	< 0.001
**Current smoker, %**	6.7	8.8	11.6	< 0.001	7.6	9.1	10.5	< 0.001
**Assessment season, %**				< 0.001				< 0.001
Winter (Jan-Mar)	25.4	25.1	24.9		25.3	25.0	24.8	
Spring (Apr-Jun)	28.7	29.3	29.5		28.5	29.2	29.3	
Summer (July-Sept)	23.1	23.9	24.3		23.3	24.1	24.6	
Fall (Oct-Dec)	22.8	21.7	21.4		22.9	21.7	21.4	
**Employment status, %**				< 0.001				< 0.001
Employed	62.6	51.0	38.1		64.4	54.6	45.8	
Retired	29.8	39.6	47.2		29.8	39.7	48.0	
Unemployed	1.3	1.5	1.6		1.1	1.2	1.2	
**Shift worker, %**				< 0.001				< 0.001
Never/Rarely	86.5	79.2	74.8		87.4	80.6	76.7	
Sometimes	6.6	8.1	8.4		6.2	7.6	7.5	
Usually	1.6	2.5	3.4		1.5	2.3	3.1	
Always	5.3	10.2	13.5		4.9	9.5	12.7	
**Excessive daytime sleepiness, %**				< 0.001				< 0.001
Never/Rarely	89.1	62.0	51.8		89.8	65.1	57.8	
Sometimes	10.4	34.3	30.0		9.8	32.0	29.4	
Often	0.5	3.7	18.2		0.4	2.9	12.8	
All the time	0.0	0.0	0.1		0.0	0.0	0.0	

Mean ± standard deviation or %. P-values derived from chi-squared or ANOVA test. A greater Townsend index score implies a greater degree of deprivation.

Supplementary Table 2. X chromosome association signals for daytime napping in participants of European ancestry from UK Biobank (*n* =452,633) and sex-stratified analyses.

						Full UK Biobank (*n* =452,633)			Male (*n* =207,108)			Female (n =245,525)			Sex-based heterogeneity
**SNP**	**Chr:position (NCBI Build 37)**	**Nearest gene(s)**	**Alleles (E/A)**	**EAF**	**Info**	**Beta**	**SE**	***P* Value**	**Beta**	**SE**	***P* Value**	**Beta**	**SE**	***P* Value**	***P* Value**
rs138274479	X:68830935	*EDA*	G/T	0.81	0.98	0.0092	0.0013	7.9E-13	0.0091	0.0017	1.5E-07	0.0099	0.0020	9.6E-07	0.76
rs4521934	X:101234773	*ZMAT1*	T/C	0.46	0.99	0.0058	0.0010	5.6E-09	0.0067	0.0014	4.8E-07	0.0040	0.0016	1.2E-02	0.20
rs144895060	X:133314686	*GPC3, CCDC160*	T/C	0.07	0.96	0.0114	0.0020	6.3E-09	0.0126	0.0026	1.6E-06	0.0099	0.0031	1.2E-03	0.50
rs6621715	X:102901207	*TCEAL1*	G/T	0.60	1.00	0.0060	0.0010	7.2E-09	0.0081	0.0014	3.2E-09	0.0024	0.0016	1.6E-01	0.006
rs6526791	X:14639460	*GLRA2*	A/T	0.43	0.98	0.0056	0.0010	4.2E-08	0.0064	0.0014	3.3E-06	0.0048	0.0016	2.3E-03	0.46

Genetic association analysis for the X chromosome was performed in related subjects of European ancestry using BOLT-LMM linear mixed models and an additive genetic model adjusted for age, sex, 10 principal components of ancestry, genotyping array and genetic correlation matrix [jl2]. Beta (SE) estimates are per each additional effect allele. Positive beta indicates more frequent daytime napping.
Abbreviations: Chr=chromosome, E/A=effect/alternative alleles, Info=imputation quality score, position=base pair coordinate hg19, SNP=single nucleotide polymorphism.

Supplementary Table 3. Lookup of previously reported daytime napping signals and other suggestive loci from studies of accelerometer-derived phenotypes related to daytime napping in present UK Biobank analysis.

						Results in present UK Biobank analysis				
**Study Population (*n*)**	**Phenotype**	**Study Pubmed ID**	**SNP**	**Chr:position (NCBI Build 37)**	**Nearest gene**	**Alleles (E/A)**	**Info**	**Beta**	**SE**	***P***
UK Biobank (*n* =386,577)	Self-reported daytime napping	30804565	rs2820313	1:201870221	*SHISA4*	G/A	1.00	0.013	0.001	**1.50E-23**
UK Biobank (*n* =386,577)	Self-reported daytime napping	30804565	rs2367277	3:192758964	*FGF21*	A/G	1.00	0.003	0.001	7.90E-03
UK Biobank (*n* =386,577)	Self-reported daytime napping	30804565	rs2653344	6:55133586	*HCRTR2*	T/C	1.00	0.017	0.001	**5.00E-29**
UK Biobank (*n* =386,577)	Self-reported daytime napping	30804565	rs34799682	9:81743116	*RP11-165H23.1*	G/A	0.99	0.014	0.002	**6.80E-21**
UK Biobank (*n* =386,577)	Self-reported daytime napping	30804565	rs2763895	13:22446756	*FGF9*	G/A	0.99	0.005	0.001	1.30E-04
UK Biobank (*n* =386,577)	Self-reported daytime napping	30804565	rs117124984	17:44051588	*RPS26P8*	C/G	0.98	0.021	0.001	**1.30E-45**
UK Biobank (*n* =386,577)	Self-reported daytime napping	30804565	rs2048524	18:44799195	*SKOR2*	G/A	0.99	0.013	0.001	**5.80E-27**
UK Biobank (*n* =85,670)	Accelerometer-derived daytime inactivity duration	30952852	rs17805200	9:13764434	*MPDZ/NFIB*	C/T	1.00	0.001	0.001	4.20E-01
UK Biobank (*n* =85,670)	Accelerometer-derived daytime inactivity duration	30952852	rs7155227	14:63365094	*KCNH5*	T/G	0.98	0.002	0.001	2.10E-01
LIFE Adult Study (*n* =956)	Actigraphic daytime resting, weekdays	27126917	rs28360899	10:125772237	CHST15	A/G	0.93	0.001	0.003	7.90E-01
LIFE Adult Study (*n* =956)	Actigraphic daytime resting	27126917	rs3800123	6:1903590	*GMDS*	T/A	0.98	0.003	0.002	2.20E-01
LIFE Adult Study (*n* =956)	Actigraphic daytimg napping duration, weekdays	27126917	rs6550704	3:22633761	*ZNF385D*	T/A	0.98	0.001	0.001	6.00E-01
LIFE Adult Study (*n* =956)	Actigraphic daytime napping	27126917	rs117097805	14:43529765	*TUBBP3*	G/A	0.99	0.002	0.003	5.60E-01
LIFE Adult Study (*n* =956)	Aactigraphic daytime resting	27126917	rs16885979	6:54625815	*FAM83B*	A/G	0.98	0.000	0.003	8.80E-01

Genetic association from present analysis was performed in related subjects of European ancestry using BOLT-LMM linear mixed models and an additive genetic model adjusted for age, sex, 10 principal components of ancestry, genotyping array and genetic correlation matrix [jl2]. Beta (SE) estimates are per each additional effect allele. Positive beta indicates more frequent daytime napping.
Abbreviations: Chr=chromosome, E/A=effect/alternative alleles, Info=imputation quality score, position=base pair coordinate hg19, SNP=single nucleotide polymorphism.

Supplementary Table 4. Descriptive table of 23andMe research participants of European ancestry (*n* =541,333) included in daytime napping replication analysis.

**Characteristic**	**Never/Rarely (*n* =267,271)**	**Sometimes (n =232,868)**	**Always (*n* =41,194)**	***P* value**
**Sex (female), n (%)**	66.3	62.1	55.8	< 0.001
**Age, y**	49.78 (15.87)	51.02 (16.94)	58.74 (17.57)	< 0.001
**Sleep duration, hours***	6.83 (1.17)	6.77 (1.21)	6.64 (1.49)	< 0.001
**BMI, kg/m2***	27.45 (6.00)	28.51 (6.45)	29.48 (6.89)	< 0.001

Mean ± standard deviation or %. P values were derived from one way ANOVA tests for continuous variables and from Chi-squared tests for binary variables. *Sleep duration missing for n=142,559; BMI data missing for n=13,497.

Supplementary Table 5. Inverse-variance weighted regression of 123 daytime napping signals on accelerometer-derived sleep measures in the UK Biobank (*n* =85,499).

Accelerometer-derived sleep measure	Beta [95% confidence interval]	*P* value
Daytime inactivity duration, minutes	0.315 [0.226, 0.404]	4.21E-12
L5 timing (midpoint of the least-active 5 h of the day), minutes	-0.082 [-0.251, 0.088]	0.35
M10 timing (midpoint of the most-active 10 h of the day), minutes	-0.037 [-0.212, 0.138]	0.68
Number of sleep bouts, *n*	0.342 [-0.235, 0.919]	0.25
Sleep duration, minutes	0.046 [-0.09, 0.183]	0.51
Sleep duration standard deviation, minutes	0.003 [-0.063, 0.069]	0.94
Sleep efficiency, %	-0.012 [-0.023, -0.001]	3.50E-02
Sleep midpoint, minutes	-0.026 [-0.096, 0.045]	0.47

Beta is effect on outcome per each additional daytime napping effect allele.

Supplementary Table 6. Genome-wide genetic correlation between daytime napping and other self-reported and accelerometer-derived sleep traits in the UK Biobank using LDSC.

Trait		r_g	*SE*	*P* value	h2_obs	h2_obs_se	h2_int	h2_int_se	gcov_int	gcov_int_se
Self-reported sleep traits	Daytime sleepiness	0.702	0.017	0.00E+00	0.049	0.002	1.017	0.009	0.343	0.007
	Long sleep duration	0.420	0.025	1.94E-64	0.030	0.002	1.013	0.007	0.153	0.006
	Sleep duration	0.204	0.023	5.54E-19	0.069	0.003	1.022	0.009	0.124	0.007
	Insomnia	0.196	0.024	4.39E-16	0.117	0.005	1.017	0.009	0.065	0.007
	Snoring	0.147	0.023	3.94E-10	0.061	0.003	1.027	0.009	0.054	0.006
	Chronotype	0.122	0.023	7.71E-08	0.111	0.004	1.042	0.013	-0.008	0.008
	Short sleep duration	-0.023	0.028	4.09E-01	0.050	0.002	1.023	0.008	-0.004	0.007
	Obstructive sleep apnea	-0.008	0.025	7.51E-01	0.206	0.023	1.000	0.007	-0.004	0.005
	Ease of awakening	0.000	0.024	9.99E-01	0.075	0.003	1.047	0.010	-0.079	0.007
Accelerometer-derived sleep traits	Daytime inactivity duration	0.346	0.029	9.48E-34	0.116	0.008	0.996	0.007	0.072	0.006
	Sleep midpoint	-0.164	0.039	2.24E-05	0.068	0.007	1.019	0.007	0.003	0.006
	Sleep efficiency	-0.117	0.031	1.56E-04	0.102	0.008	1.010	0.007	-0.036	0.006
	L5 timing	-0.135	0.038	3.39E-04	0.082	0.007	1.017	0.007	0.007	0.006
	M10 timing	-0.124	0.039	1.49E-03	0.066	0.007	1.013	0.007	-0.016	0.005
	Sleep duration standard deviation	0.138	0.052	8.62E-03	0.030	0.006	1.006	0.007	0.014	0.005
	Number of sleep bouts	0.030	0.027	2.65E-01	0.176	0.009	1.009	0.008	0.014	0.006
	Sleep duration	0.003	0.028	9.24E-01	0.145	0.010	1.007	0.008	-0.021	0.006

Supplemental Table 7. Multi-trait colocalization of daytime napping with self-reported sleep traits in the UK Biobank.

Colocalized trait with daytime napping	Posterior probability of colocalization	Regional association probability	Candidate variant	CADD score	Posterior probability explained by variant	Chr	Nearest gene
Daytime sleepiness	0.9834	1.0000	rs13284688	22	0.6012	9	*RP11-165H23.1*
Daytime sleepiness	0.9812	0.9999	rs285793	0.57	0.4353	8	*ZFPM2*
Daytime sleepiness	0.9662	0.9803	rs614987	2	0.4144	6	*NKAIN2*
Ease of awakening, chronotype	0.9576	1.0000	rs2653349	19.56	0.6711	6	*HCRTR2*
Daytime sleepiness	0.9572	0.9633	rs351776	6.22	0.6631	8	*PNOC*
Daytime sleepiness	0.9345	0.9940	rs62519825	8.26	0.0966	8	*RP11-21C4.5*
Daytime sleepiness	0.9306	0.9931	rs9965170	0.54	0.1272	18	*SKOR2*
Daytime sleepiness	0.9231	1.0000	rs13010456	0.63	0.1103	2	*AGAP1*
Sleep duration	0.9174	0.9923	rs34354917	0.49	0.5059	12	*ALG10B*
Daytime sleepiness, sleep duration	0.9055	0.9999	rs1846644	0.89	0.8674	12	*KSR2*
Daytime sleepiness, sleep duration	0.8944	0.9577	rs147880726	1.02	0.5628	1	*RP11-740P5.3*
Daytime sleepiness	0.8857	0.9617	rs224111	0.03	0.2261	10	*RP11-436D10.3*
Sleep duration	0.8787	1.0000	rs8050478	2.91	0.1193	16	*RP11-461O7.1*
Snoring	0.8775	0.9918	rs2224195	4.41	0.1781	6	*SNAP91*
Daytime sleepiness	0.8755	0.9327	rs9389556	21.8	0.3296	6	*RP3-359N14.1*
Daytime sleepiness	0.8753	0.9989	rs60579048	0.6	0.0300	1	*KIAA1522*
insomnia	0.8651	0.9960	rs4916720	6.25	0.0633	5	*RPS3AP22*
Chronotype	0.8579	0.9350	rs10257273	1.33	0.3127	7	*GS1-259H13.13*
Sleep duration	0.8316	0.9796	rs4587762	0.15	0.1949	11	*DRD2*
Insomnia, chronotype	0.8281	1.0000	rs77960	0.26	0.4821	5	*RP11-6N13.1*
Chronotype	0.8261	0.9205	rs2250377	20.7	0.1493	1	*SHISA4*
Daytime sleepiness	0.8241	0.8505	rs1843815	2.51	0.9861	1	*NFU1P2*
Ease of awakening, chronotype	0.8151	0.9977	rs1400280	1.72	0.2492	12	*CPNE8*
Daytime sleepiness, snoring, chronotype	0.8138	0.9200	rs12140153	26.1	1.0000	1	*PATJ*
Daytime sleepiness	0.8079	0.8809	rs553314	10.42	0.9786	1	*RP5-827O9.1*
Daytime sleepiness	0.8018	0.8387	rs2390669	2.05	0.5266	2	*STK39*
Daytime sleepiness	0.8018	0.8284	rs10875622	2.12	0.4818	5	*STK32A*
Chronotype	0.8008	0.9960	rs786406	0.53	0.2978	2	*CAMKMT*
Sleep duration	0.7976	0.9957	rs11224896	2.36	0.0536	11	*TRPC6*
Sleep duration	0.7752	0.9112	rs7900191	1.52	0.1597	10	*PDZD8*
Daytime sleepiness	0.7347	0.8404	rs60222088	4.87	0.2664	12	*RP11-554D14.1*
Daytime sleepiness	0.7157	0.8284	rs10150432	2.16	0.1117	14	*RP11-562L8.1*

Abbreviations: CADD=Combined Annotation Dependent Depletion; Chr=chromosome.

Supplemental Table 8. Multi-trait colocalization of daytime napping variants with body mass index.

Posterior probability of colocalization	Regional association probability	Candidate variant	CADD score	Posterior probability explained by variant	Chr	Nearest gene	Concordant effect on higher BMI and increased daytime napping?
0.9927	1.0000	rs2481665	9.54	0.9086	1	*PATJ*	Yes
0.9696	0.9825	rs3914188	2.86	0.9372	3	*ECE2*	Yes
0.9667	0.9974	rs7503597	0.37	0.9095	17	*BAIAP2*	No
0.9627	1.0000	rs2820311	8.42	0.2217	1	*IPO9*	Yes
0.9489	0.9713	rs16826068	1.04	0.9276	1	*MACF1*	Yes
0.9410	1.0000	rs12022461	15.71	0.2293	1	*KIAA1522*	Yes
0.9305	0.9997	rs853676	10.89	0.4323	6	*ZSCAN31*	No
0.9255	0.9946	rs7187776	3.83	0.5745	16	*TUFM*	Yes
0.9148	0.9906	rs2023671	1.04	0.5398	16	*PRKCB*	Yes
0.9093	0.9999	rs3843540	1.11	0.2532	7	*ZKSCAN5*	Yes
0.8723	0.9741	rs17167306	1.77	0.1161	7	*EXOC4*	Yes
0.8596	0.8818	rs11721853	4.01	0.7488	4	*LNCPRESS2*	No
0.8584	0.9959	rs166195	1.49	0.1704	3	*MRAS*	No
0.8560	0.8596	rs17207196	2.42	0.9953	7	*POM121C*	Yes
0.8377	0.9826	rs351776	6.22	0.5844	8	*PNOC*	Yes
0.8371	0.9980	rs273697	1.6	0.1744	18	*RN7SL97P*	-
0.8134	0.8522	rs785145	4.51	0.2413	6	*HS3ST5*	-
0.7810	0.8362	rs8059538	0.36	0.7254	16	*AC010546.1*	-
0.7506	0.8200	rs17817449	15.33	0.9430	16	*FTO*	-

Abbreviations: CADD=Combined Annotation Dependent Depletion; Chr=chromosome.

Supplementary Table 9. Colocalization of daytime napping variants with frontal cortex gene expression (GTEXv7, *n* =129).

Posterior probability of colocalization	Regional association probability	Candidate variant	CADD score	Posterior probability explained by variant	Chr	Gene name	GTEx *P* Value
0.988	1.000	rs1001817	5.74	0.262	3	*ECE2*	6.62E-09
0.888	1.000	rs174561	13.14	0.263	11	*FADS1*	5.47E-08
0.877	0.996	rs12024415	0.54	0.609	1	*CCDC190*	9.45E-09
0.873	0.990	rs1048015	0.35	0.472	22	*IFT27*	3.07E-11
0.754	0.995	rs6452787	0.05	0.168	5	*ENSG00000271904*	5.78E-07
0.746	0.995	rs12493586	8.14	0.054	3	*ZNF660*	2.85E-09

Abbreviations: CADD=Combined Annotation Dependent Depletion; Chr=chromosome.

Supplementary Table 10. Tissue enrichment analysis of gene expression in GTEx RNA-seq data using MAGMA. Significant tissue enrichments with P <9.43E-04 for 53 tested tissues (=0.05/53) are bolded.

Tissue	Observed Genes	Beta	SE	*P* Value
**Brain_Frontal_Cortex_BA9**	**16805**	**0.0466**	**0.0090**	**1.18E-07**
**Brain_Nucleus_accumbens_basal_ganglia**	**16805**	**0.0512**	**0.0099**	**1.26E-07**
**Brain_Cortex**	**16805**	**0.0459**	**0.0093**	**3.54E-07**
**Brain_Anterior_cingulate_cortex_BA24**	**16805**	**0.0471**	**0.0095**	**3.79E-07**
**Brain_Hypothalamus**	**16805**	**0.0507**	**0.0105**	**7.60E-07**
**Brain_Caudate_basal_ganglia**	**16805**	**0.0472**	**0.0104**	**2.69E-06**
**Brain_Cerebellum**	**16805**	**0.0368**	**0.0082**	**3.99E-06**
**Brain_Cerebellar_Hemisphere**	**16805**	**0.0354**	**0.0080**	**4.73E-06**
**Brain_Amygdala**	**16805**	**0.0430**	**0.0105**	**1.99E-05**
**Brain_Putamen_basal_ganglia**	**16805**	**0.0427**	**0.0105**	**2.32E-05**
**Brain_Hippocampus**	**16805**	**0.0413**	**0.0105**	**4.28E-05**
**Brain_Substantia_nigra**	**16805**	**0.0398**	**0.0115**	**2.85E-04**
Pituitary	16805	0.0237	0.0113	0.02
Brain_Spinal_cord_cervical_c-1	16805	0.0222	0.0115	0.03
Esophagus_Muscularis	16805	0.0047	0.0162	0.39
Esophagus_Gastroesophageal_Junction	16805	0.0027	0.0168	0.44
Adrenal_Gland	16805	-0.0029	0.0127	0.59
Nerve_Tibial	16805	-0.0036	0.0141	0.60
Colon_Sigmoid	16805	-0.0056	0.0162	0.64
Ovary	16805	-0.0047	0.0127	0.64
Testis	16805	-0.0040	0.0069	0.72
Muscle_Skeletal	16805	-0.0078	0.0091	0.81
Uterus	16805	-0.0131	0.0150	0.81
Skin_Sun_Exposed_Lower_leg	16805	-0.0096	0.0102	0.83
Cells_Transformed_fibroblasts	16805	-0.0098	0.0097	0.84
Skin_Not_Sun_Exposed_Suprapubic	16805	-0.0113	0.0102	0.87
Bladder	16805	-0.0179	0.0159	0.87
Artery_Tibial	16805	-0.0167	0.0133	0.89
Cervix_Endocervix	16805	-0.0205	0.0158	0.90
Cervix_Ectocervix	16805	-0.0256	0.0174	0.93
Thyroid	16805	-0.0198	0.0132	0.93
Heart_Atrial_Appendage	16805	-0.0225	0.0126	0.96
Heart_Left_Ventricle	16805	-0.0209	0.0116	0.96
Cells_EBV-transformed_lymphocytes	16805	-0.0121	0.0067	0.96
Artery_Aorta	16805	-0.0272	0.0137	0.98
Pancreas	16805	-0.0226	0.0112	0.98
Esophagus_Mucosa	16805	-0.0222	0.0099	0.99
Small_Intestine_Terminal_Ileum	16805	-0.0257	0.0110	0.99
Vagina	16805	-0.0352	0.0149	0.99
Prostate	16805	-0.0374	0.0155	0.99
Artery_Coronary	16805	-0.0391	0.0158	0.99
Colon_Transverse	16805	-0.0348	0.0139	0.99
Fallopian_Tube	16805	-0.0434	0.0162	1.00
Whole_Blood	16805	-0.0204	0.0075	1.00
Adipose_Subcutaneous	16805	-0.0405	0.0142	1.00
Minor_Salivary_Gland	16805	-0.0371	0.0129	1.00
Liver	16805	-0.0242	0.0082	1.00
Breast_Mammary_Tissue	16805	-0.0525	0.0166	1.00
Kidney_Cortex	16805	-0.0396	0.0122	1.00
Lung	16805	-0.0423	0.0126	1.00
Stomach	16805	-0.0557	0.0154	1.00
Spleen	16805	-0.0352	0.0095	1.00
Adipose_Visceral_Omentum	16805	-0.0548	0.0146	1.00

MAGMA analysis conditioned on gene size, log gene size, gene density, log gene density, inverse minor allele count, and log inverse minor allele count.

Supplementary Table 11. Characteristics of genome-wide association study used for daytime napping polygenic risk score (123 loci) associations and two-sample Mendelian randomization.

					Daytime napping polygenic score associations		
**Trait**	**Trait unit**	**Sample size (*n* total or *n* cases/*n* controls)**	**GWAS consortium, Pubmed ID**	**Ancestry**	**Beta**	***SE***	***P* Value**
BMI	SD kg/m2	322,154	GIANT, 25673413	European	0.191	0.049	1.04E-04
Waist circumference	SD cm	232,101	GIANT, 25673412	European	0.279	0.056	6.94E-07
Waist-hip-ratio adjusted for BMI	SD	210,082	GIANT, 25673412	European	0.182	0.055	9.76E-04
LDL cholesterol	SD mg/dL	173,082	GLGC, 24097068	Predominantly European	-0.233	0.086	6.71E-03
HDL cholesterol	SD mg/dL	187,167	GLGC, 24097068	Predominantly European	-0.347	0.079	1.15E-05
Triglycerides	SD mg/dL	177,861	GLGC, 24097068	Predominantly European	0.173	0.077	2.49E-02
Fasting glucose	mmol/L	58,074	MAGIC, 22581228	Predominantly European	0.019	0.041	6.42E-01
Fasting insulin	log pmol/L	51,750	MAGIC, 22581228	Predominantly European	0.106	0.042	1.25E-02
HOMA-B	log HOMA	46,186	MAGIC, 20081858	European	0.123	0.044	5.53E-03
HOMA-IR	log HOMA	46,186	MAGIC, 20081858	European	0.156	0.054	3.63E-03
HbA1c	%	46,368	MAGIC, 20858683	European	-0.031	0.046	4.97E-01
Diastolic blood pressure	mmHg	337,054	UKB	European (White British) – non-overlapping with current analysis	1.126	0.345	1.08E-03
Systolic blood pressure	mmHg	337,054	UKB	European (White British) – non-overlapping with current analysis	3.724	0.513	3.80E-13
Coronary artery disease	log-odds	60,801/123,504	CARDIoGRAMplusC4D, 26343387	Predominantly European	0.146	0.110	1.85E-01
Type 2 diabetes	log-odds	26,488/83,964	DIAGRAM, 24509480	Predominantly European	0.252	0.226	2.65E-01

Beta is effect on outcome per each additional daytime napping effect allele.

Supplementary Table 12. Mendelian randomization sensitivity analyses for effect of daytime napping on outcomes.

Method	Exposure	Outcome	*n* SNPs	Beta [95% CI]	P value
MR-PRESSO	Nap_1	DBP_2	101	2.63 [1.42, 3.85]	5.01E-05
	Nap_1	SBP_2	103	3.23 [1.06, 5.40]	4.30E-03
	Nap_2	DBP_1	98	2.73 [1.41, 4.05]	1.00E-04
	Nap_2	SBP_1	100	2.69 [0.41, 4.98]	2.30E-02
	Nap	Waist circumference	73	0.28 [0.12, 0.43]	8.30E-04
Multivariable MR	Nap_1	DBP_2	101	2.69 [1.17, 4.21]	5.46E-04
	Nap_1	SBP_2	103	3.15 [0.46, 5.85]	2.00E-02
	Nap_2	DBP_1	98	1.92 [0.38, 3.46]	1.48E-02
	Nap_2	SBP_1	100	2.99 [0.40, 5.59]	2.39E-02
	Nap	Waist circumference	73	0.16 [-0.02, 0.35]	8.59E-02

"Nap" refers to the genetic instrument generated using the entire UKB sample.
Nap_1 and Nap_2 refer to the estimates generated using the split-sample MR approach.
Multivariable MR estimates have been adjusted for pleiotropic associations of the variants with short sleep duration and with insomnia.

Supplementary Table 13. Characteristics of genome-wide association study samples used for *HCRTR1* and *HCRTR2* phenome-wide association analyses.

Trait	Trait unit	Contributing datasets or consortia*	Pubmed ID	*n* total or *n* cases /*n* controls
Restless legs syndrome	log-odds	23andme, EU-RLS GENE, INTERVAL	29029846	45,896 / 382,683
Coronary artery disease	log-odds	UK Biobank, CARDIoGRAM+C4D	29212778	122,733 / 424,528
Atrial fibrillation	log-odds	UK Biobank, AFGen	30061737	60,620 / 970,216
Any ischemic stroke	log-odds	ISGC	29531354	34,217 / 406,111
Large artery stroke	log-odds	ISGC	29531354	4,373 / 301,63
Small vessel stroke	log-odds	ISGC	29531354	5,386 / 348,946
Cardioembolic stroke	log-odds	ISGC	29531354	7,193 / 362,661
Body mass index	SD kg/m2	GIANT, UK Biobank	30239722	806,834
Waist-hip-ratio adjusted for BMI	SD	GIANT, UK Biobank	30239722	697,734
Systolic blood pressure	mmHg	ICBP, UK Biobank	30224653	~750,000
Diastolic blood pressure	mmHg	ICBP, UK Biobank	30224653	~750,000
Pulse pressure	mmHg	ICBP, UK Biobank	30224653	~750,000
Pulse rate	SD beats per minute	UK Biobank	31768069	430,029
White matter hyperintensity volume	SD mm3	UK Biobank	31768069	12,065
Smoking initiation	SD of weighted average prevalence in meta-analysis	Tobacco and Genetics Consortium, UK Biobank	30643251	557,337 / 674,754
Type 2 diabetes	log-odds	DIAGRAM, UK Biobank	30054458	62,892 / 596,424
LDL cholesterol	SD mg/dL	GLGC	24097068	173,082
HDL cholesterol	SD mg/dL	GLGC	24097068	187,167
Triglycerides	SD mg/dL	GLGC	24097068	177,861
