# Supplementary material for: Genetic determinants of daytime napping and effects on cardiometabolic health
Source: Nat Commun. 2021 Feb 10;12:900. doi: 10.1038/s41467-020-20585-3 (PMC7876146; doi:10.1038/s41467-020-20585-3)
Supplement: Supplementary file 3 — Reporting Summary [file 41467_2020_20585_MOESM3_ESM.pdf]

nature research

Corresponding author(s): Richa Saxena

Last updated by author(s): 12/07/2020

Reporting Summary

Nature Research wishes to improve the reproducibility of the work that we publish. This form provides structure for consistency and transparency in reporting. For further information on Nature Research policies, see our Editorial Policies and the Editorial Policy Checklist.

Statistics

For all statistical analyses, confirm that the following items are present in the figure legend, table legend, main text, or Methods section.

n/a	Confirmed	
☐	☒	The exact sample size (n) for each experimental group/condition, given as a discrete number and unit of measurement
☐	☒	A statement on whether measurements were taken from distinct samples or whether the same sample was measured repeatedly
☐	☒	The statistical test(s) used AND whether they are one- or two-sided<br>*Only common tests should be described solely by name; describe more complex techniques in the Methods section.*
☐	☒	A description of all covariates tested
☐	☒	A description of any assumptions or corrections, such as tests of normality and adjustment for multiple comparisons
☐	☒	A full description of the statistical parameters including central tendency (e.g. means) or other basic estimates (e.g. regression coefficient) AND variation (e.g. standard deviation) or associated estimates of uncertainty (e.g. confidence intervals)
☐	☒	For null hypothesis testing, the test statistic (e.g. F, t, r) with confidence intervals, effect sizes, degrees of freedom and P value noted<br>*Give P values as exact values whenever suitable.*
☒	☐	For Bayesian analysis, information on the choice of priors and Markov chain Monte Carlo settings
☐	☒	For hierarchical and complex designs, identification of the appropriate level for tests and full reporting of outcomes
☐	☒	Estimates of effect sizes (e.g. Cohen's d, Pearson's r), indicating how they were calculated

Our web collection on statistics for biologists contains articles on many of the points above.

Software and code

Policy information about availability of computer code

Data collection	No data collection
Data analysis	We primarily used PLINK, R, BOLT-REML, METAL, FUMA, and LDSC. Other software was used as described in the methods section.

For manuscripts utilizing custom algorithms or software that are central to the research but not yet described in published literature, software must be made available to editors and reviewers. We strongly encourage code deposition in a community repository (e.g. GitHub). See the Nature Research guidelines for submitting code & software for further information.

Data

Policy information about availability of data

All manuscripts must include a data availability statement. This statement should provide the following information, where applicable:

- Accession codes, unique identifiers, or web links for publicly available datasets
- A list of figures that have associated raw data
- A description of any restrictions on data availability

This research has been conducted using the UK Biobank Resource (UK Biobank application number 6818) and 23andMe. Data is available through the central UK Biobank study, which accepts applications.

Field-specific reporting

Life sciences study design

All studies must disclose on these points even when the disclosure is negative.

Sample size	Sample size was achieved as described in Methods section on lines 584 and 639.
Data exclusions	Exclusion criteria were preestablished and include subjects of non-European ancestry (as determined by genetic principle components), subjects withdrawn from the study, samples/SNPs failing quality control, or with missing or extreme phenotypic values (described in methods).
Replication	We replicated our findings within our discovery cohort in the 23andMe study. Results were consistent across all replication attempts.
Randomization	Randomization was not relevant as study was observational.
Blinding	Blinding was not relevant as study was observational.

Reporting for specific materials, systems and methods

We require information from authors about some types of materials, experimental systems and methods used in many studies. Here, indicate whether each material, system or method listed is relevant to your study. If you are not sure if a list item applies to your research, read the appropriate section before selecting a response.

Materials & experimental systems

n/a	Involved in the study	
☒	☐	Antibodies
☒	☐	Eukaryotic cell lines
☒	☐	Palaeontology and archaeology
☒	☐	Animals and other organisms
☐	☒	Human research participants
☒	☐	Clinical data
☒	☐	Dual use research of concern

Methods

n/a	Involved in the study	
☒	☐	ChIP-seq
☒	☐	Flow cytometry
☒	☐	MRI-based neuroimaging

Human research participants

Policy information about studies involving human research participants

Population characteristics	Among UK Biobank participants of European ancestry (n =452,633), 38.2% and 5.3% of participants reported sometimes and always napping, respectively (Supplementary Table 1). Overall, participants reporting always napping were more likely to be older males, report longer 24h sleep duration, have higher body mass index (BMI), waist circumference, systolic and diastolic blood pressures, have a higher Townsend deprivation index (i.e., greater degree of socio-economic deprivation), report being current smokers, unemployed or retired, and shift workers (all P <0.001; Supplementary Table 1). The Mass General Brigham is a hospital-based cohort study from the Mass General Brigham hospitals in Boston, MA with electronic health record (EHR) and genetic data. Recruitment for the Mass General Brigham launched in 2010 and is active at participating clinics at Brigham and Women's Hospital, Massachusetts General Hospital, Spaulding Rehabilitation Hospital, Faulkner Hospital, McLean Hospital, Newton-Wellesley Hospital, and North Short Medical Center.
Recruitment	The UK Biobank is a large population-based study established to allow detailed investigations of the genetic and lifestyle determinants of a wide range of phenotypes58. Data from >500,000 participants living in the United Kingdom who were aged 40-69 and living <25 miles from a study center participated in the study between 2006 and 2010. 23andMe, Inc. is a personal genetics company.
Ethics oversight	The UK Biobank study was approved by the National Health Service National Research Ethics Service (ref. 11/NW/0382). Approval for analysis of Mass General Brigham Biobank data was obtained by Mass General Brigham IRB, protocol #2018P002276. 23andMe analyses were reviewed and approved by Ethical & Independent Review Services, a private institutional review board (http://www.eandireview.com).

Note that full information on the approval of the study protocol must also be provided in the manuscript.
