# Supplementary material for: Genetic determinants of daytime napping and effects on cardiometabolic health
Source: Nat Commun. 2021 Feb 10;12:900. doi: 10.1038/s41467-020-20585-3 (PMC7876146; doi:10.1038/s41467-020-20585-3)
Supplement: Supplementary file 4 — Description of Additional Supplementary Files [file 41467_2020_20585_MOESM4_ESM.pdf]

Description of Additional Supplementary Files

Supplementary Data 1. Genome-wide association signals for daytime napping in participants of European ancestry from UK Biobank (n =452,633) and subsequent sensitivity analyses.

Supplementary Data 2. Replication and meta-analysis of genome-wide association signals for daytime napping in UK Biobank (n =452,633) with 23andMe research participants (n =541,333).

Supplementary Data 3. Association of genome-wide association signals for daytime napping with accelerometer-derived sleep measures in the UK Biobank.

Supplementary Data 4. Association of genome-wide association signals for daytime napping with other self-reported sleep traits in the UK Biobank.

Supplementary Data 5. Functional annotation of genome-wide association signals for daytime napping.

Supplementary Data 6. Convergence of daytime napping variants' clustering using Bayesian nonnegative matrix factorization (bNMF) algorithm to 3 likely distinct daytime napping-promoting mechanisms.

Supplementary Data 7. Gene-based association analysis using Pascal (summing statistics across independent signals in each gene) for daytime napping.

Supplementary Data 8. MAGMA pathway-based enrichment analysis based on MAGMA gene-sets. All 15,481 pathways are shown.

Supplementary Data 9. Pascal pathway-based enrichment test using Pascal (gene-set enrichment analysis using 1,077 pathways from KEGG, REACTOME, BIOCARTA databases). Significant pathways with P <4.64E-05 for 1,077 tested pathways are bolded.

Supplementary Data 10. Genetic correlation between daytime napping and 257 traits using LD-score regression from publicly available genome-wide association study data. For each model (BMI unadjusted and adjusted), significant correlations with P <1.9E-04 accounting for 257 tested traits are bolded.

Supplementary Data 11. Association between genome-wide polygenic score for daytime napping and diseases from phenome-wide association study (951 diseases) in the Partners Biobank (n =23,561).

Supplementary Data 12. Mendelian randomization sensitivity analyses for effect of daytime napping on outcomes.

Supplementary Data 13. Sample sizes for each phecode category used in phenome-wide association analysis. Data obtained from the Lee Lab website: https://www.leelabsg.org/resources.
